# forceplate: An R package for processing raw force-plate time-series data

**DOI:** 10.3758/s13428-025-02657-8

**Published:** 2025-06-03

**Authors:** Raphael Hartmann, Anton Koger, Elisa R. Straub, Leif Johannsen, Iring Koch, Denise N. Stephan, Hermann Müller, Andrea Kiesel

**Affiliations:** 1 University of Freiburg, Freiburg, Germany; 2 University of Aachen, Aachen, Germany; 3 University of Gießen, Gießen, Germany

**Keywords:** R package, Force plate, Time series, Segmentation, Event locking, Event related

## Abstract

Evidence supporting the interaction between cognitive and motor processes is increasing. Conventional approaches to analyze balance control aggregate sway data over seconds up to minutes, which presents a challenge in discerning the impact of single cognitive processes on balance control. In this paper, we propose a novel, event-related approach to investigate how cognitive task performance affects balance control on small time scales using a force plate. A force plate continuously measures forces and moments in each spatial dimension over time. To facilitate the processing of the resulting time-series data, we developed an R-package called forceplate. This package segments the data so that each trial, corresponding to a cognitive task, has its own time-series data. A low-pass filter can be applied to remove artifacts (e.g., muscle twitches or electrical noise), and a baseline correction can be performed to improve the comparability of trials. For each trial’s time-series data, user-defined descriptive statistics (e.g., mean or standard deviation) can be calculated for user-defined time bins around an event (e.g., stimulus or response onset). The package generates a dataset with one or more measures per trial (depending on the number of time bins) that can be used for further analysis, such as a (mixed-effects) analysis of variance. The R-package and the described underlying procedure aim to establish a standard to process force-plate data collected in the context of cognitive experiments for the event-related approach. This facilitates the processing of force-plate data and enhances comparability between studies.

Psychological research often conceptualizes cognitive and motor processes as unrelated. For example, in cognitive psychology, the theoretical framework of processing stages (Sternberg, 1998) suggests that cognitive (“central”) processing stages are distinct from motor stages (see Sanders, 1998), making direct interactions between cognitive and motor processes unlikely. However, research in sports and movement sciences provides abundant evidence of a strong influence of cognition, emotion, and motivation on motor control (e.g., Rosenbaum, 2010). Despite this, the exact mechanisms underlying this influence on motor control often remain somewhat underspecified.

This issue is particularly relevant in the context of research on age-related motor deficits, where the increased risk of falls is a significant concern (see e.g., Melzer et al., 2001). It has been shown that some age-related motor deficits are not purely motoric but are also due to age-related cognitive changes in attentional control (Woollacott and Shumway-Cook, 2002; see also Boisgontier et al., 2013). However, pinpointing the exact mechanisms by which cognitive factors specifically affect motor control is challenging.

One important research tool for examining psychological factors in motor control is posturography (Duarte & Freitas, 2010; Mouras et al., 2023; Paillard & Noé, 2015; Visser et al., 2008; Winter, 1995). Often, body balance and balance control while standing is assessed by using force plates, which measure body sway through ground-reaction force dynamics (Duarte & Freitas, 2010). The stability of body sway can be analyzed using several measures (Raymakers et al., 2005), such as variability measures like the standard deviation or root mean square of center-of-pressure (CoP) displacement or CoP velocity over a given time period (often ranging from 5 s to 12 s, or even up to 30 s; see e.g., Kang and Lipsitz, 2010; Kerr et al., 1985; VanderVelde et al., 2005; Vuillerme et al., 2000; Latash et al., 2003). The CoP is a critical concept used to describe the point on a supporting surface where the resultant pressure (or force) is applied. This point shifts as the body moves and changes balance on a mediolateral-axis (i.e., side-to-side movements) and an anteroposterior-axis (i.e., the front-to-back movements) providing insight into balance, stability, and overall movement control (Winter, 1995). While the measure of the standard deviation of the CoP indicates how much the data deviate from its mean value, the root mean square of CoP displacement measures the average amplitude of CoP movements over time. The velocity of the CoP is the rate at which the center of pressure moves across the support surface. Analyzing the CoP is utilized in clinical diagnostics and rehabilitation, sports science, and ergonomics to assess and improve balance, stability, movement efficiency, and injury prevention (for a review see Schubert, 2014). Interference between cognitive tasks and balance control during standing has been well documented (e.g., Caron et al., 2020; Melzer et al., 2001; Redfern et al., 2018; Stephan et al., 2018; Straub et al., 2022), but is beyond the scope of the current work.

However, balance control measures aggregated over longer time periods (i.e., blocks of trials) do not allow for the examination of potentially subtle time-locked cognitive-motor interactions. There are reasons why such aggregated measures of balance control have been used in the past; for example, aggregating over longer time periods decouples the time-variable cognitive processes preceding an observed motor response from the slower balance oscillations (Petrigna et al., 2021). Additionally, aggregation across longer time periods can account for issues of biokinetic inertia (e.g., Bottaro et al., 2008), which refers to the inherent resistance of a biological system to changes in motion or direction, supporting the maintenance of balance. However, similar considerations apply to neural measures of brain activity, such as in electrophysiology (e.g., EEG data) or in the inertia of the hemodynamic response when measuring changes in regional blood flow (e.g., the BOLD response). This research has benefited tremendously from new methodological approaches that allow for more “real-time” assessment using *event-related*, trial-based designs (see D’Esposito et al., 1999; Luck et al., 1990). The implementation of an event-related approach in measurements of balance control (i.e., providing highly time-resolved information about the CoP) allows for the detection of detailed changes in balance and precise adaptations of balance control processes.^1^ This method yields important and complementary information that is being masked in measurements over longer periods due to a body’s physical properties such as the aforementioned biokinetic inertia.

In a recent study, Johannsen et al. (2023) introduced an event-related, trial-based (rather than block-based) approach to examine the influence of attentional control in a cognitive task on balance control during upright standing, with a sub-second temporal resolution of 150-ms time bins. As a cognitive task, they used the ”Simon” task, which examines how irrelevant spatial information interferes with response selection (Simon, 1969). Participants respond to a stimulus based on certain attributes (such as color or shape), but the spatial location of the stimulus (left or right) can conflict with the required response location (usually a left or right manual response). It was demonstrated that resolving response conflict (see Hommel, 2011, for a review) in a ”Simon” task reduces variability in balance control in the mediolateral-axis prior to the manual response compared to trials without this conflict.

The variability of the force moments (sway) within a time bin reflects the amount of neuromuscular activity devoted to maintaining standing, with higher variability indicating a greater likelihood of balance adjustments being made within that time frame (Gawthrop et al., 2011). Johannsen et al. (2023) argued that the conflict-induced delay of response selection in the ”Simon” task may result in an overlap of capacity-limited cognitive processes (required for resolution of response conflict and selection of a manual response) and intermittent control impulses responsible for balance corrections. The concept of intermittent control impulses suggests that balance control occurs in discrete bursts or impulses (e.g., Gawthrop et al., 2011). Assuming that response conflict in incongruent conditions requires suppression of incorrect response tendencies, as reviewed by Cespón et al. (2020), their results suggest that these cognitive mechanisms of conflict resolution have downstream consequences for balance control mechanisms. The reduction in sway variability suggests a “micro-bottleneck” which omits or delays intermittent control impulses that would have been triggered close in time to the response-selection process. Importantly, these interactions between cognitive and balance control were not observed in previous studies due to lack of temporal precision of traditional methods that aggregate data over longer intervals. This underscores the relevance of the new event-related, trial-based approach.

As with all innovative developments, there is no tradition and thus no established procedure to assess and analyze the corresponding continuous force-plate data. Moreover, a common issue in many fields of research is the variability in data processing techniques, which can significantly affect the outcomes of analyses. This has also been demonstrated in the processing of force-plate data where different techniques can lead to inconsistent results (Rhea et al., 2015). Additionally, the derivation of the most relevant dependent measures depends on experimenter decisions and possibly other somewhat arbitrary factors. Additionally, handling the typically massive amount of data, combined with the continuous time-series nature of these data, can be very challenging and time-consuming for individual researchers. Therefore, a ready-to-use software package representing a well-developed and validated analytical tool for employing event-related designs with a force plate would be highly valuable. It would allow researchers from different labs to process their data in a highly comparable way and could expedite scientific advancements in the field of cognitive influences on motor control. The primary objective of the package is to standardize an analysis procedure to ensure the production of reproducible results in the research field.

In the present paper, we present such a comprehensive software package, implemented as an R package (R Core Team, 2023), called forceplate. This package allows researchers to process raw force-plate data in a principled and standardized way. It is based on the procedures established by Johannsen et al. (2023), but it incorporates additional flexibility to accommodate different choices and adjustments. In their study, all raw force-plate data were processed using MATLAB (The MathWorks Inc., 2022) routines. For the purposes of this paper, we demonstrate how accurately these processed data can be replicated using our R package.

The outline of this paper is as follows: First, we describe the relevance of a coherent procedure for processing raw force-plate data and provide a step-by-step guide for such a procedure. We then introduce our developed R package forceplate, which facilitates these processing steps, and provide additional R code for optional steps. Afterward, we describe the study and raw force-plate data from Johannsen et al. (2023) and demonstrate how to reproduce its processing steps using our R package. We then compare the resulting processed dataset from the R package with the processed data from the original study, where MATLAB was used. Finally, we summarize the paper and discuss our scientific contribution, the reproducibility of the published processed data, and some limitations. Additionally, we created material on how to set up laboratories, which can be found in the Supplemental Materials (https://osf.io/ebzrv/).

## Method

The force-plate data consists of time-series measurements of force and moment, typically captured at a high frequency (i.e., 1000 Hz or higher). These measurements allow for detailed analysis of balance, stability, and movement dynamics by capturing the subtle shifts in pressure and force exerted by the body during standing. To connect this data to cognitive processes in an experiment, an event-related approach is necessary. Before introducing our recommended standard procedure for processing raw force-plate time-series data, it is important to explain what an event-related approach entails and what it demands from the data.

### Event-related approach

An event-related approach focuses on specific time bins within the time-series data (e.g., a 150-ms segment of moment measurements along the mediolateral-axis) that are temporally linked to certain reoccurring events in an experiment (e.g., pressing a response button under a specific condition). Notably, the timeframe of 150 ms as well as the spatial information (i.e., mediolateral-axis and anteroposterior-axis) are adjustable. For example, in conflict tasks, cognitive conflict as well as control processes can be associated with specific time periods after stimulus presentation, respectively, and can be individually examined by adjusting the timeframe (Nieuwenhuis & de Kleijn, 2013; Ridderinkhof, 2002). The key interest lies in summarizing these time bins using descriptive statistics (such as mean, standard deviation, or range). In the context of force plates, these statistical summaries help determine whether systematic differences exist in the selected time bin, which might indicate the influence of cognitive processes on balance control (similar to event-related approaches in EEG or fMRI; see e.g., Hu and Zhang, 2019).

However, to effectively link the time-series data to specific events, the data must be ”enriched” with markers – referred to as triggers – that identify the relevant time segments. Typically, experiments are organized into trials, with each trial corresponding to an experimental condition. Various triggers might be used to mark the onset of key events, such as the appearance of a fixation cross, the presentation of a stimulus, the moment a response is made, the condition under which the participant is operating, and whether the response was correct, among others. These triggers, or a combination of them, might encode the experimental conditions. Additionally, in more complex experimental setups where multiple tasks are performed within a single trial, the triggers need to clearly indicate the respective events for each task.

This approach requires that the triggers are transmitted, often via a parallel port (see Supplemental Materials on https://osf.io/ebzrv/ for details on how to transmit triggers), to the device that records the raw force-plate data.^2^ An example of the technical specifics of setting up and installing such a system are also explained in the Supplemental Materials.

### Procedure

In the following, we describe processing steps proposed by Johannsen et al. (2023) to prepare force-plate data for analysis. The required steps are (1) filtering, (2) segmentation, (3) baseline correction, and (4) calculating (descriptive) statistics of interest for time-locked bins per trial. These trial-level statistics can then be used for subsequent statistical analyses, which are beyond the scope of this paper.

#### Filtering

The first step in processing force-plate time-series data is to apply filters to minimize artifacts. Winter (2009, Chapter 3 & 10) recommended using a low-pass Butterworth filter for human movement data, noting that movement artifacts typically range from about 10 Hz (e.g., muscle twitches) to 1000 Hz (white noise from electromagnetic signals). For instance, Johannsen et al. (2023) applied a fourth-order low-pass Butterworth filter with a cut-off at 10 Hz, effectively reducing electromagnetic noise. To minimize phase lags and edge artifacts, they employed a customized forward-backward (or zero-phase) filtering technique; instead of simply applying the filter, reversing the time-series, reapplying the filter, and reversing the time-series again, they appended the first *n* (in this case, 2000) data points in reverse order each time before applying the filter, then removed these points afterward. We recommend adopting this procedure to effectively reduce artifacts.

#### Segmentation

After the low-pass filtering, the time-series data need to be segmented so that each segment corresponds to a single trial. This means each trial has its own dedicated time-series data. Segmentation of force-plate time-series data requires event markers. This necessitates that each change during the experiment (e.g., presentation of the fixation cross, stimulus onset, participant response) is encoded in the raw force-plate data through signals from the experimental program. These signals should be transmitted via a parallel port. The number of pins ($N_{pin}$) in the parallel port determines the number of possible signals ($2^{N_{pin}}$) that can be transmitted. We recommend continuously sending a signal until the next event in the experiment triggers a new signal (see Supplemental Material for detailed explanations). Using the onset of the fixation cross as a marker for trial segmentation is a natural choice but is not the only option. At this point, one might also generate new variables by transforming existing ones.

#### Baseline correction

To make the time-series data across trials more comparable, we suggest applying a baseline correction (see e.g., Kirchner et al., 2012; Schubert et al., 2012a; Schubert et al., 2012b). Rather than subtracting the mean of the entire time series within a trial, we recommend subtracting the mean of a baseline interval (e.g., the fixation-cross interval) within each trial (Johannsen et al., 2023), since this is a phase without cognitive or muscular demands.

#### Statistics of time-locked bins

Once the time-series data are segmented (and baseline corrected), Johannsen et al. (2023) proposed defining time-locked bins and calculating descriptive statistics such as the mean or standard deviation within each bin. This involves defining bins in milliseconds relative to specific events (e.g., onset of stimulus, response, or inter-trial interval) – sometimes referred to as event-locked bins – and then aggregating the data within these bins. Note that it is also possible to extract multiple time bins before and after the specific event and calculate descriptive statistics for each time bin individually. These descriptive statistics for each trial can be used in further analyses (e.g., as dependent variables in an analysis of variance).

### R Package

*R* (R Core Team, 2023) is a programming language designed for statistical computation, with the advantage of being free and open-source. One of its strengths is the ease with which users can share their own functions by developing R packages and making them available on CRAN (Comprehensive R Archive Network).

**Fig. 1:**
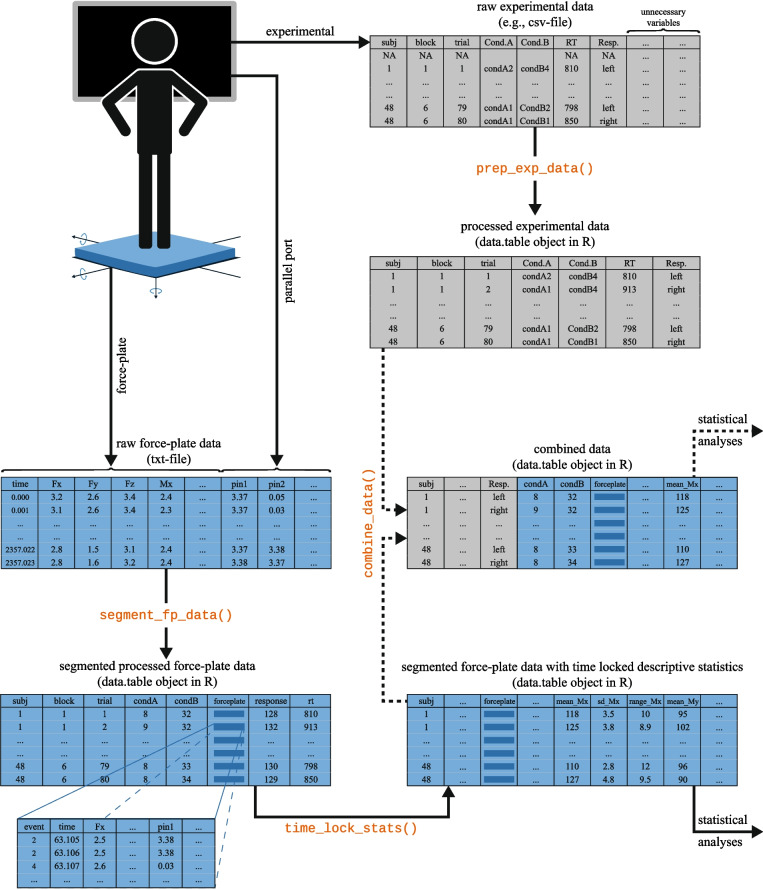
Schema for processing the raw force-plate data and the raw experimental data

Below, we outline the functions and processing steps in the order we suggest they be performed. Figure 1 provides a schematic overview of the processing steps along with the corresponding R functions from the forceplate package.

#### Requirements

Before using the package, certain requirements must be met. First, each data file’s name must include the string *subj* followed by the subject number, which identifies the subject. If data are saved in separate files per block, the filename should also include the string *block* followed by the block number (e.g., exp1_fp_subj001_block001.txt would be an acceptable file name). This is particularly crucial for the force-plate data, as unlike experimental data, force-plate data usually do not contain subject, block, or trial information.^3^ Consequently, this information will be automatically saved in the processed force-plate data. Second, the filenames of all experimental data (i.e., the data produced by the experimental programming tool) must also contain the string *subj* followed by the subject number and, if applicable, the string *block* followed by the block number (e.g., exp1_subj001.csv would be a valid file name for a file that contains all blocks).

There are multiple manufacturers for force plates and each might have their own idiosyncratic file formats. To address this situation, the package uses an accessible file format: text file (.txt). These files can consist of a header with specific information about the manufacturer, force plate, sample frequency, date etc. (which can be skipped when segmenting the data). The main body should be formatted as a DSV (delimiter-separated values) file that uses a specific character, such as a comma, tab, or semicolon, to separate individual data fields within each line. Each line represents one measurement point while each data field (or column) represents a value written by the force plate or some type of DAQ (data acquisition system). There are three types of data: (1) time, (2) force-plate measures of interest like Fx, and (3) pins. The time can be an ascending number for example in milliseconds. Pins contain the current status (voltage) of each of the pins of the parallel port.

#### Installation

The R package forceplate can be installed like any other R package within the CRAN repository via install.packages(”forceplate”) (R Core Team, 2023). Alternatively, the package is available on GitHub at https://github.com/RaphaelHartmann/forceplate and can be installed using the command devtools::install_github(”RaphaelHartmann/forceplate”) (using the R package devtools by Wickham et al., 2022).

#### Processing experimental data

When working with one or more experimental datasets (e.g., behavioral data from tools like E-prime) for each participant, one can use the prep_exp_data() function to automatically clean up the datasets by removing unnecessary rows and columns. This function also combines all the datasets into one large dataset. The output of prep_exp_data() is a data.table from the R package data.table (Dowle & Srinivasan, 2023), which allows for efficient data manipulation by reference. The function arguments can be found in Appendix A.

#### Filtering force-plate data

The package includes the option to apply a fourth-order low-pass Butterworth filter (see the next paragraph). If one plans to use a different low-pass filter or another type of filter, one can do so using the R package signal (Ligges et al., 2023). The filters implemented in signal are compatible with those available filters that can be used in MATLAB/Octave. More about how to apply filters before using our package can be found in Appendix A.

#### Processing force-plate data – Segmentation

To segment the force-plate data into individual trials, one can use the segment_fp_data() function. It works with multiple datasets, and the output will be one large combined dataset. The output of segment_fp_data() is a large data.table, where the time-series data for each trial is stored within a data.table nested inside the actual |data.table|. These nested data.tables are particularly convenient for storing more than one scalar within a single data cell. The function arguments can be found in Appendix A.

#### Adding variables

If you need to add variables to the force-plate data after segmentation, you can do so using data.table’s style of adding variables. An example on how to add a variable to the existing data is in Appendix A.

#### Processing force-plate data – Time-locking

To calculate statistics (such as the mean or standard deviation) for specific bins around a time lock in the segmented time-series data, one can use the time_lock_stats() function. The output of time_lock_stats() is the same data.table as the input, but with the calculated statistics for the specified bins and variables stored in new columns. The function arguments can be found in Appendix A.

#### Combination of experimental and force-plate data

After processing is complete, one can use combine_data() to merge two experimental data tables from prep_exp_data(), combine two force-plate data tables from segment_fp_data() or time_lock_stats(), or combine experimental and force-plate data tables into a single data.table. More about combining data can be found in Appendix A.

#### Outlier handling

Once all data processing is complete, one can begin cleaning the data by excluding participants and trials based on one’s criteria for statistical analysis. For examples on how to exclude participants or trials, see Appendix A.

### Validation

To validate our R package, we reproduced the processing steps of an existing study and compared our processed data to the processed data from that study. For this, we used the raw force-plate data from Johannsen et al. (2023). In their study, participants performed a ”Simon” task while either standing or sitting (within-person condition). For our purposes, we focused on the raw data from all participants while standing. The processed data, along with the R script used to process the raw data, are available at https://osf.io/ebzrv/.

The experiment by Johannsen et al. (2023) was divided into ten blocks, resulting in ten text files per subject generated by BioWare (Kistler Group, 2012). Each block consisted of 80 trials (i.e., n.trials = 80). Baseline correction was performed by subtracting the mean of the pre-trial fixation interval (approximately 200 ms) from all data points within that trial. The actual fixation intervals varied between 214 and 250 ms (but almost all fixation intervals were either 216 or 217 ms), and the trigger used for the fixation was 128 (i.e., baseline.trigger = 128 and baseline.intv = c(0, 215)).^4^ Each trial began with a fixation cross, so the onset of the fixation cross was used to mark the start of each trial (i.e., start.trigger = 128). One of the two stimuli was presented either on the left or right side of the screen, leading to four stimulus conditions, which were coded with triggers 1, 2, 4, and 8 (i.e., stimulus.trigger.list = c(1,2,4,8)). Responses were coded as correct or incorrect with triggers 32 or 64, respectively (i.e., response.trigger.list = c(32,64)). These triggers were also used to get the conditions (i.e., cond.trigger.list) for stimulus and position (i.e., stimulus) and correctness (i.e., correctness). For the other arguments in the segment_fp_data() function, we used the defaults, which align with the procedures described by Johannsen et al. (2023) and BioWare (Kistler Group, 2012). For instance, they used a fourth-order low-pass Butterworth filter with a 10-Hz cut-off frequency, which is the default setting in our R-package.

Johannsen et al. (2023) focused on two measurements, *Mx* and *My* (i.e., vars = c(”Mx”, ”My”)), which refer to the plate moments along the mediolateral and anteroposterior axes, respectively, in newton-meters. We limited our comparison to the response time-lock (i.e., time.lock.trigger = c(32,64)). Specifically, we were interested in the measurements within two time bins: 150 ms before and 150 ms after stimulus onset (i.e., bins = c(-150,150) and bin.width = 150). The mean, standard deviation, and range of these measurements within each bin were calculated (i.e., using the default of the FUN argument).

The corresponding code with the two main functions is shown below:
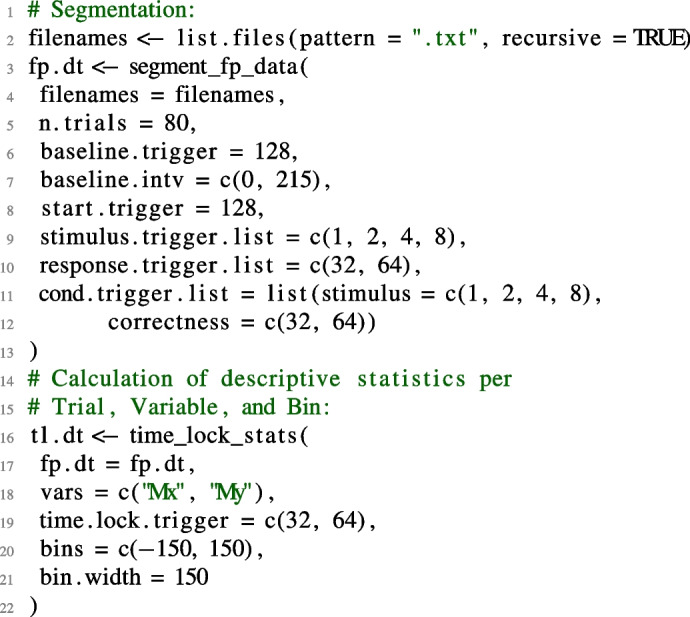


We used the R package rmatio (Widgren & Hulbert, 2023) to import the resulting MATLAB files from Johannsen et al. (2023) into R. We then excluded all trials that Johannsen et al. (2023) had excluded and split the remaining trials into four conditions: *left-location left-target*, *right-location left-target*, *left-location right-target*, and *right-location right-target*. Finally, we calculated the differences between the processed MATLAB data and our results for the mean, standard deviation, and range statistics.

## Results

Table 1 presents the five-point summary of the differences (per trial) in the resulting statistics (mean, standard deviation, and range) for the plate moments in the mediolateral- and anteroposterior-axis (Mx and My) between the study by Johannsen et al. (2023) and our R package forceplate. As the table shows, the differences are minimal, with median differences ranging, in absolute terms, between 1.474e-11 and 4.612e-13. The first and third quartiles of the differences are largest for the mean statistics, with absolute values between 6.528e-04 and 3.397e-04. For the standard deviation and range, the absolute values of the first and third quartiles fall between 6.662e-11 and 3.552e-12. The maximum differences, in absolute terms, range between 0.219 and 0.108.

**Table 1:** Five-point summary statistics of the differences between the processed data by Johannsen et al. (2023) and our R package for each combination of descriptive statistic and variable

stats	vars	min	$1^{st}$ quart.	median	$3^{rd}$ quart.	max
mean	Mx	-0.154	-3.847-04	-2.359e-12	3.397e-04	0.108
mean	My	-0.219	-6.528e-04	-4.612e-13	6.413e-04	0.185
sd	Mx	-1.658e-02	-9.829e-12	-2.341e-12	3.552e-12	4.171e-03
sd	My	-1.010e-07	-2.196e-11	-4.074e-12	1.139e-11	5.908e-10
range	Mx	-4.467e-01	-3.020e-11	-8.042e-12	8.989e-12	8.470e-03
range	My	-5.294e-07	-6.662e-11	-1.474e-11	2.978e-11	2.965e-09

*Note.* Five-point summary statistics of the difference between the processed data by Johannsen et al. (2023) and our R package for each statistic (”stats”) and variable (”vars”) combination, where ”sd” denotes the standard deviation, and ”Mx” and ”My” denote the moment (in newton-meter) on the mediolateral-axis and anteroposterior-axis, respectively, on the force plate. ”min” denotes the minimum, ”$1^{st}$ quart.” the first quartile, ”$3^{rd}$ quart.” the third quartile, and ”max” the maximum difference. We use the scientific notation for very small differences. For example, $-1.658e-02$ means $-1.658\times 10^{-2}$

**Fig. 2:**
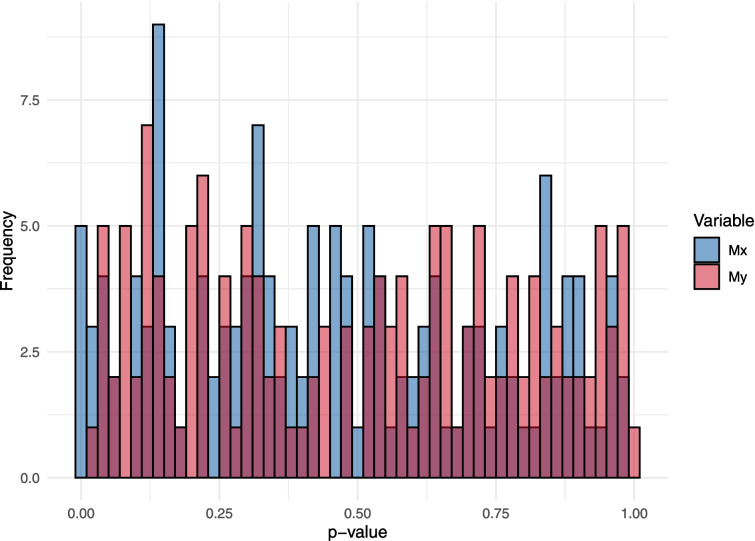
Histogram of the *p* values from paired *t* tests comparing the descriptive statistics calculated from MATLAB and our R package

As shown, the mean statistic exhibits the greatest variance among the three descriptive statistics (see first and third quartiles in Table 1). This variance is largely due to the slightly different baseline correction methods used (see Appendix B). Baseline correction is just subtracting the mean of the fixation period from all values in a time series. Johannsen et al. (2023) used the entire fixation period (mostly 216 or 217 ms per trial) as the basis for baseline correction, while we used a fixed 216 ms for each trial. The mean of these corrected measures is more affected than the standard deviation or range due to its higher sensitivity to the values on which it is calculated. Another, albeit less significant, source of the differences could be the differences in the implementation of some basic routines.^5^

However, these differences between the two approaches are negligible. In fact, the *p* values from paired *t*-tests (comparing descriptive statistics calculated in MATLAB vs. the forceplate package) for each combination of subject, variable (Mx and My), and descriptive statistic do not significantly differ from a uniform distribution according to a Kolmogorov-Smirnov test for uniformity ($D(288)=0.063$, $p=.201$; see also Fig. 2 for a histogram of the *p* values). Nonetheless, understanding the sources of variance in the differences is valuable (see Appendix B for more details). Most of the differences in the mean statistic occur at the fourth decimal place or higher, while the differences in the other statistics typically occur at the eleventh to twelfth decimal places or beyond. This level of precision reinforces our confidence in the correct implementation of the algorithms.

**Fig. 3:**
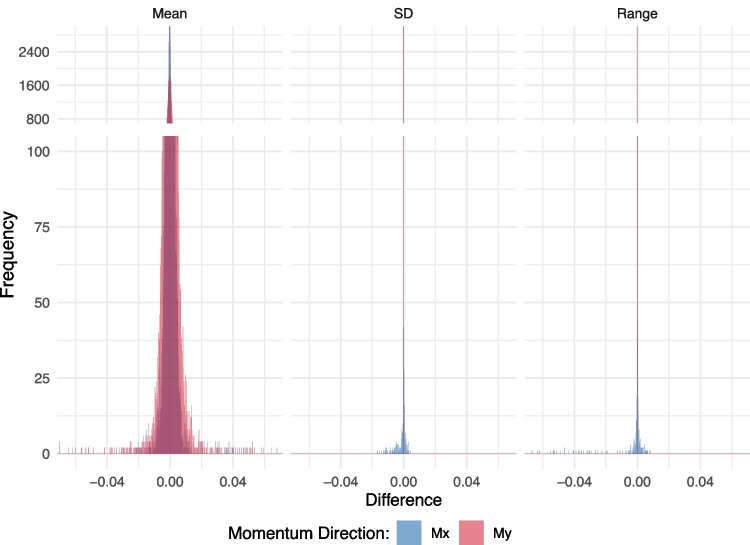
Histogram of the differences between the processed data by Johannsen et al. (2023) and our R package for each combination of descriptive statistic and variable when using response time-lock

Figure 3 shows the distribution of the differences between the two approaches. It clearly illustrates that the differences are minimal, with only a few outliers.

## Discussion

The goal of this manuscript was to introduce a possible standard procedure for processing raw force-plate data, specifically by segmenting the data into trials and calculating event-related descriptive statistics. Within this processing framework, it is possible to temporally align specific balance events on the force plate with corresponding cognitive events. To achieve this, we developed an R package called forceplate, which simplifies the processing of raw force-plate data. To validate this software, we processed the raw force-plate data from Johannsen et al. (2023) using our developed software package and compared the resulting processed data to the one by Johannsen et al. (2023), thereby establishing a reproducible standard procedure.

By providing a standardized method for processing raw force-plate data, we aim to enhance the use of force plates in research on cognitive-motor interactions. This allows researchers to focus more on formulating their research questions and analyzing the processed data, rather than on developing their own data processing procedures and then implementing them in a programming language. Additionally, using the same or similar procedures across different studies can enhance the comparability of analysis results.

Even though we provided recommendations for processing raw force-plate data, such as applying a fourth-order low-pass Butterworth filter with cut-off frequency of 10 Hz and using a baseline correction, it is not the only way of processing these data. The low-pass Butterworth filter was chosen as the conventional method often applied to kinesiological data (e.g., Winter, 2009) due to the filter’s properties offering a good compromise regarding the advantages and disadvantages of a continuous signal filter (Robertson & Dowling, 2003). Johannsen et al. (2023) used a cut-off frequency of 10 Hz. According to Nashner (1977) situational balance adjustments take at least 100 ms, which is why 10 Hz seemed an appropriate choice for us. The choice for the baseline correction was rather pragmatic; it was used by Johannsen et al. (2023). Nevertheless, there are other ways of processing the raw data. In Appendix A we provide code to circumvent the implemented filter and baseline correction and use custom ones instead.

Our R package forceplate is free and open-source, enabling users to easily segment raw force-plate data into trials and calculate descriptive statistics for event-related time bins and variables for further analysis. It offers options such as filtering and baseline correction, making it adaptable to the user’s needs. Additionally, it preserves all data points (i.e., the time-series data) for each trial, including all triggers.

Moreover, the package supports open science and reproducibility. By providing a processing script (or just the inputs of the arguments) along with the raw force-plate data (and specifying the package version), users can ensure that the processing steps are reproducible.

The reproduction of the processed force-plate data from Johannsen et al. (2023) confirmed that our R package is correctly implemented. The differences between the two approaches (MATLAB and our R package) were negligible and likely stemmed from slight procedural variations and rounding differences between the programming languages.

The R-package is highly adaptable to various research needs: (1) Different experimental conditions only require that the relevant conditions are encoded in triggers.^6^ If this requirement is met and the data can be exported to a text file, our R-package can be used to process the data; (2) different force-plate settings, such as sampling frequency, can be accommodated simply by specifying the appropriate value in the corresponding function argument; (3) different cognitive tasks may require different intervals for baseline corrections and different triggers, all of which can be specified in the function arguments. It is even possible to handle tasks that consist of multiple sub-tasks.

With our R package forceplate it is possible to process raw time-series data from force plates. However, it is important to note that we did not cover how to use the resulting processed data to run statistical analyses. Our focus is solely on the processing of raw force-plate data. That said, once the data has been processed, it is straightforward to use the final dataset to run, for example, an analysis of variance in R using the aov() function. Even hierarchical data analysis is feasible, as the resulting datasets include observations for each trial within each subject.

Enabling researchers to examine the impact of cognitive processes on balance control in a fine-grained, event-related manner will hopefully contribute to a deeper understanding of the interplay between cognitive and motor control processes. This, in turn, may facilitate future research on important societal challenges, such as addressing the consequences of aging populations. For example, forceplate could prove useful in better understanding the relationship between cognitive distraction and increased fall risk in elderly individuals, ultimately aiding in the prevention of falls among the elderly.

## Figures and Tables

**Fig. 4:**
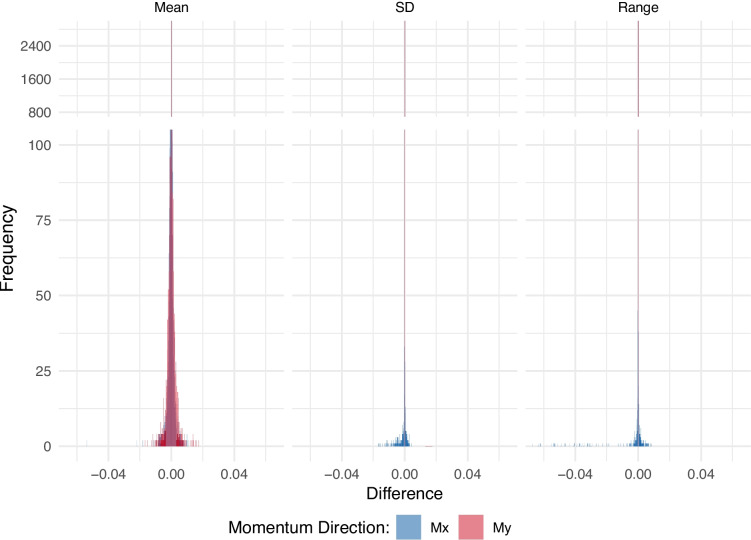
Histogram of the differences between the processed data by Johannsen et al. (2023) and our R package for each combination of descriptive statistic and variable when using response time-lock

## Data Availability

The R code for this project can be found on OSF at https://osf.io/ebzrv/.

## Appendix A Use of the R package

### Processing experimental data

When working with one or more experimental datasets (e.g., behavioral data from tools like E-prime) for each participant, one can use the prep_exp_data() function to automatically clean up the datasets by removing unnecessary rows and columns. This function also combines all the datasets into one large dataset. The function has six arguments:

- filenames must be a character vector containing the paths to the experimental datasets. As mentioned earlier, the filenames must include the string *subj* followed by the subject ID (a number). If the data is saved in separate files per block, the filenames must also include the string *block* followed by the block number. For example, ”path/to/Experiment1_subj5_block2.csv” would be a valid element of filenames.
- na.strings must be a character vector specifying the strings to be interpreted as NA (not available). For instance, you might set it to c(”,,”, ”[]”) to treat ”,,” and ”[]” as missing entries in the datasets.
- excl.vars must be a character vector providing the names of the variables that should be used to remove rows from the datasets. For example, if it is set to c(”trial”, ”rt”, ”condition”), a row will be removed from a dataset if all corresponding cell entries in these three columns are NA. Therefore, it is crucial to set the na.strings argument correctly.
- blacklist.vars and whitelist.vars must be character vectors used to filter unnecessary columns or variables from the datasets. Only one of these arguments needs to be specified. Use blacklist.vars to explicitly exclude the named variables, or whitelist.vars to include only the named variables.
- sort must be set to either TRUE or FALSE. If set to TRUE, all files will be sorted by subject ID and, if applicable, block number.

The output of prep_exp_data() is a data.table from the R package data.table (Dowle & Srinivasan, 2023), which allows for efficient data manipulation by reference.

### Filtering force-plate data

The package includes the option to apply a fourth-order low-pass Butterworth filter (see the next paragraph). If one plans to use a different low-pass filter or another type of filter, one can do so using the R package signal (Ligges et al., 2023). The filters implemented in signal are compatible with those available filters that can be used in MATLAB/Octave.

For example, a fourth-order low-pass Butterworth filter can be applied in a different way. Here, before applying the filter we prepend the first 2000 (except the very first) data points in reverse order, append the last 2000 (except the very last) data points in reversed order. Then we apply the filter and remove the added data points. In the package, this is done in two steps (i.e., the filter is applied two times, once with the data points in the original order and once in reversed order but always with additional data points to reduce artifacts).

### Processing force-plate data – segmentation

To segment the force-plate data into individual trials, one can use the segment_fp_data() function. It works with multiple datasets, and the output will be one large combined dataset. The function has 15 arguments:

- filenames is similar to the prep_exp_data() function, but here it should include the paths to the force-plate text files. The naming convention for these text files must follow the same pattern as the experimental datasets (as described above).
- n.trials must be an integer indicating the number of trials per dataset. If the number of trials varies across datasets, n.trials must be a vector of integers (matching the length of filenames) that specifies the number of trials for each dataset. For example, if an experiment consists of two blocks with 60 and 80 trials, respectively, stored in two separate files (Experiment1_subj1_block1.txt and Experiment1_subj1_block2.txt), and one wants to segment both files in one step, then n.trials must be set to c(60, 80).
- start.trigger and start.prepend define the segmentation boundaries for the trials. start.trigger must be a vector of trigger numbers that mark trial onsets (e.g., the onset of the fixation cross trigger). Each trial will include the data points from this onset to one data point before the next onset, or to the last data point if it is the final trial in the dataset. If one wants to include a buffer before each trial onset, one can set start.prepend to a value greater than zero, which will prepend extra data points to each trial, effectively shifting the actual trial onset to the specified number of milliseconds before the trigger onset (e.g., before the fixation cross).
- baseline.trigger and baseline.intv are used for baseline correction within each trial. This requires setting triggers in baseline.trigger, where the onset of these triggers serves as a reference point (i.e., zero) for the interval defined in baseline.intv. For example, if baseline.intv = c(-199, -100) and the trigger for the fixation cross is used, an interval from 199 to 100 ms *before* the fixation-cross onset would be defined. If baseline.trigger = 0, no baseline correction is applied.
- stimulus.trigger.list and response.trigger.list specify the trigger numbers used for stimuli and responses, respectively. These arguments can either be a vectors or named lists of vectors. If a trial involves only one task (the most common case), a vector of trigger numbers is sufficient. However, if a trial includes multiple tasks (e.g., an auditory and a visual task), a named list must be used to differentiate between tasks (e.g., stimulus.trigger.list = list(visual = c(8,9), auditory = c(16,18))). The same applies to response triggers. These arguments are necessary for calculating response times (RTs) for each task from the force-plate data, which can then be compared to RTs from the experimental dataset.
- cond.trigger.list must be a named list. For each name, a variable will be stored indicating the condition (which can be compared to the condition in the experimental dataset).
- variable.names must be a named list of variable names. There are three types of variables: (1) time, which must be set to the name of the time variable used in the force-plate data (e.g., time = ”abs time (s)” in BioWare by Kistler Group, 2012; 2) variables for all pins in the parallel port, which should be named using pin followed by a number from 1 to 8 (e.g., pin1) and matched to the names used in the force-plate data (e.g., in BioWare, all pins are named aux, so pin1 = ”aux”, pin2 = ”aux”, etc., with the function using the order in which pin1 to pin8 are provided); and (3) other variables, such as any potential measures of interest like Fx. There are no restrictions on naming these variables; for example, one might rename Fx from the force-plate data to Fx = ”x-Force” or any other preferred name.
- skip must be an integer indicating the number of lines to skip in each force-plate data file. Typically, the first few lines of a data file contain software and settings information. To access the data, these lines must be skipped when reading the file. The default is set to 19, which is the number of lines typically used by BioWare Kistler Group (2012) for such information.
- az0 must be a negative integer representing the thickness parameter of the force plate in millimeters. This argument is only necessary if the center of pressure in the *x* and *y* directions should be calculated. Otherwise, the default value of zero can be used.
- sampling.freq specifies the sampling frequency in Hz.
- cutoff.freq specifies the cut-off frequency for the fourth-order Butterworth filter. If set to zero, no filter will be applied.
- imputation provides an option for imputing missing values in the force-plate data. Usually, this is not needed, so it is set to NULL by default. However, if required, it can be set to one of the following: ”fmm”, ”periodic”, ”natural”, ”monoH.FC”, or ”hyman”, which are options of the spline() function from the stats package (R Core Team, 2023).
- sort must be set to either TRUE or FALSE. If set to TRUE all files in filenames are sorted by subject ID and, if applicable, block number.

The output of segment_fp_data() is a large data.table from the R package data.table (Dowle & Srinivasan, 2023), where the time-series data for each trial is stored within a data.table nested inside the actual data.table. These nested data.tables are particularly convenient for storing more than one scalar within a single data cell.

### Baseline correction

In case you prefer to use your own baseline correction, you can do so by first applying the segment_fp_data() function without baseline correction (i.e., with the argument baseline.trigger = 0) and then apply your own baseline correction on the resulting object from segment_fp_data(). Here is an example on how to do so:

The custom baseline correction bs.correction() takes exactly the number of data points from the fixation-cross period (the baseline correction in segment_fp_data() takes a fixed interval for each trial), calculates the mean of these points and subtracts it from all other values in the trial. This is a more exact approximation to the procedure by Johannsen et al. (2023).

### Adding variables

If you need to add variables to the force-plate data after segmentation, you can do so using |data.table|’s style of adding variables. For example, if you want to multiply variable My by 1000 and store the result in a new variable My_new, you can use the following code: where fp.dt is the output from the segment_fp_data() function, and forceplate is the variable that contains all the force-plate data points for each trial. The invisible() function is used to suppress the output in the R console.

### Processing force-plate data – time-locking

To calculate statistics (such as the mean or standard deviation) for specific bins around a time lock in the segmented time-series data, one can use the time_lock_stats() function. This function has seven arguments:

- fp.dt must be the output from segment_fp_data() (i.e., a nested data.table).
- vars must be a vector containing either the column numbers or names of the variables for which the time lock should be applied.
- time.lock.trigger must be a vector of integers representing the triggers that will be used as references for the bins of interest. The onset of these triggers serves as the reference data point.
- bins must be a vector of length two or a list of many vectors of length two, specifying the bin intervals in milliseconds. For example, if one is interested in two 150-ms bins around the time lock reference, one would set bins to list(c(-149, 0), c(1, 150)).
- bin.width and n.bins are optional arguments for creating multiple bins of equal length. These options are only available if bins is a single vector of length two, which can then be divided into bins of width bin.width or into n.bins bins.
- sampling.freq specifies the sampling frequency in milliseconds.
- FUN is a list of functions to be applied to the bins of the selected variables. The functions should be summary statistics that return a scalar. By default, the mean, standard deviation, and range are calculated.

The output of time_lock_stats() is the same data.table as the input, but with the calculated statistics for the specified bins and variables stored in new columns.

### Combination of experimental and force-plate data

After processing is complete, one can use combine_data() to merge two experimental data tables from prep_exp_data(), combine two force-plate data tables from segment_fp_data() or time_lock_stats(), or combine experimental and force-plate data tables into a single data.table. For the first two options, the two data.tables are combined vertically (i.e., stacked). In the third option, the tables are combined horizontally (i.e., side by side). For the first two options, the arguments dt1 and dt2, which represent the two data.tables to be stacked, are sufficient. For the third option, there are two additional arguments. First, the by argument, which should be a list specifying the column names for the subject, block, and trial in the experimental data. Second, the continuous argument, which is a logical value indicating whether the trial numbering is continuous across blocks (i.e., sequentially numbered from one to the total number of trials) or not (i.e., resets for each block).

### Outlier handling

Once all data processing is complete, one can begin cleaning the data by excluding participants and trials based on ones criteria for statistical analysis. For example, to exclude data from subjects 5 and 12 from the data.table named exp1.dt, one could use the following code: One can also exclude specific rows by their row number (e.g., rows 99 and 201) with the following code:  For more details on working with data.table, see its package help files.

## Appendix B Raw force-plate data processing closer to Johannsen et al. (2023)

Table 2 shows the same five-point summary statistics as Table 1 with the difference that here a processing procedure for the raw force-plate data was conducted using forceplate which is more complicated but more similar to the original procedure by Johannsen et al. (2023). The baseline correction was applied exactly as in Johannsen et al. (2023), that is, we ran the function segment_fp_data() first without baseline correction and then applied the baseline-correction by hand (see Appendix A). As can be seen, the first and third quartile of the differences for the mean statistics are much closer to zero than in Table 1.

**Table 2:** Same five-point summary statistics of the differences as in Table 1 but where the baseline correction is exactly done as in Johannsen et al. (2023)

stats	vars	min	$1^{st}$ quart.	median	$3^{rd}$ quart.	max
mean	Mx	-5.351e-02	-2.819e-11	-2.735e-13	2.808e-11	1.382e-02
mean	My	-1.670e-02	-5.099e-11	9.073e-13	5.307e-11	1.755e-02
sd	Mx	-1.658e-02	-9.829e-12	-2.341e-12	3.552e-12	4.172e-03
sd	My	-1.010e-07	-2.196e-11	-4.074e-12	1.139e-11	5.908e-10
range	Mx	-4.467e-01	-3.020e-11	-8.042e-12	8.989e-12	8.470e-03
range	My	-5.295e-07	-6.662e-11	-1.474e-11	2.979e-11	2.965e-09

*Note.* Five-point summary statistics of the difference between the processed data by Johannsen et al. (2023) and our R package for each statistic (”stats”) and variable (”vars”) combination. Check the note of Table 1 for the notation. Here a processing procedure of the raw force-plate data was used that is more complicated to do with forceplate but which is more close to the original processing steps by Johannsen et al. (2023). The most noticeable differences to Table 1 are written in red

Figure 4 shows the improved difference between the two approaches graphically. The differences in the mean statistic are now more in line with the other two statistics (see Fig. 3 for the comparison).

To further investigate the differences, we used the same procedure, but this time we examined the stimulus time-lock.^7^ Table 3 shows the five-point summary statistics of the differences. There were still some outliers but the majority of differences were also on the eleventh (or higher) digit. This indicates in total that the R package aligns with the processing steps in MATLAB, but with some minor differences that are due to chance (or due to some slightly differences in how the programming languages operate).

**Table 3:** Same five-point summary statistics of the differences as in Table 2 but for stimulus time-lock

stats	vars	min	$1^{st}$ quart.	median	$3^{rd}$ quart.	max
mean	Mx	-0.096	-9.304e-12	-3.671e-14	9.108e-12	0.066
mean	My	-0.042	-1.980e-11	1.310e-13	2.030e-11	0.058
sd	Mx	-0.059	-1.101e-11	-2.568e-12	3.931e-12	0.031
sd	My	-0.034	-2.417e-11	-4.610e-12	1.241e-11	0.033
range	Mx	-0.451	-3.351e-11	-8.798e-12	9.914e-12	0.077
range	My	-0.086	-7.117e-11	-1.552e-11	3.232e-11	0.086

*Note.* Five-point summary statistics of the difference between the processed data by Johannsen et al. (2023) and our R package for each statistic (”stats”) and variable (”vars”) combination. Check the note of Table 1 for the notation. Here a processing procedure of the raw force-plate data was used that is more complicated to do with forceplate but which is more close to the original processing steps by Johannsen et al. (2023). The most noticeable differences to Table 1 are written in red

## Footnotes

1 More details on this approach can be found in Subsection “Event-related approach”.

2 Our R package requires signals through a parallel port, but it could potentially be adapted to other interfaces.

3 *Force-plate data* refers to the measurements from the force plate, including forces across the three spatial dimensions. *Experimental data* refers to the data typically collected in cognitive experiments, such as responses, response times, condition types, trial numbers, and block numbers. For a graphical representation of these data types, see Fig. 1.

4 Specifying an exact interval for each trial was not possible due to the R-package’s limitation of applying a single interval (in milliseconds) across all trials.

5 For example, 2.5 would be rounded to 2 in MATLAB and to 3 in R.

6 Unfortunately, not all force plate systems on the market support the transmission of triggers.

## Availability of data and materials

The processed data in R, materials, and preprint for this project can be found on OSF at https://osf.io/ebzrv/.
